# Reduced plantar sensation leads to heterogeneous reactions in plantar pressure distribution during normal walking

**DOI:** 10.1186/1757-1146-7-S1-A50

**Published:** 2014-04-08

**Authors:** Justin S Lange, Thomas L Milani

**Affiliations:** 1 Technische Universität Chemnitz, Chemnitz, Germany

## Introduction

Many studies have determined the influence of provoking reduced plantar foot sensitivity on plantar pressure distribution patterns during the roll-over process (ROP) [1-3], but differ considerably in their approaches and results. This raises the question of whether the method of provoking decreased plantar foot sensitivity is responsible for the different results or whether subjects respond so differently that there is no uniform ROP reaction.

Therefore, the aim of this study was to evaluate individual response patterns in the ROP after provoking reduced plantar foot sensitivity and to consider the homogeneity of this reaction pattern within the sample.

## Methods

The plantar foot of 19 subjects was treated with EMLA^®^ cream containing the active ingredients lidocaine and prilocaine [4,5]. For each subject, the plantar sensations of vibration and touch at heel, forefoot and hallux were measured before the intervention and at three intervals of 45 minutes (+15 min measuring time). Thereby the active course of the cream was documented. Nine anatomical sub-areas were identified on the peak pressure footprint [6]. The average ROP of 10 steps was determined at each measurement. Regression analyses were used to estimate the relationships among sensation and ROP variables. Using data from a control group, *'clinical significance'*[7] was used to evaluate individual subject reactions. A hierarchical cluster analysis was used to form groups with similar behaviour within the sample.

## Results

**Figure 1:**
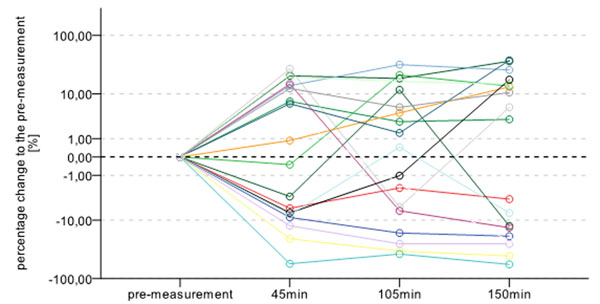
Percentage change of relative load under the first metatarsal head of each subject

Results showed strong interindividual differences in the process of sensation reduction over time. A linear relationship between change in sensory perceptions and plantar pressure variables was not detected. Nevertheless, the ROP results observed for each measurement differed strongly between and within subjects (e.g. Figure 1). Using cluster analysis, a group with a forefoot load increase was detected. Another group showed less variation in their forefoot pressure variables.

## Discussion

The heterogeneity in the responses shows that subjects react differently when plantar foot sensitivity is perturbed by EMLA^®^ cream. Thereby the ROP reaction seems to be not linear dependent from the level of reduction of plantar sensation. Indications for the existence of similar response patterns could be found despite the small sample size. In this study only a small time period of treatment was analysed. It remains unclear what would happen if plantar sensitivity was reduced for a longer period.

The complex interaction between the body, nervous system and environment may lead to various adaptive behaviours [8]. The idea of plasticity and modular control of locomotor patterns [9] could be useful for further interpretations.
